# Integrated physiological, photochemical and anatomical responses reveal divergent drought-response strategies among herbaceous peony cultivars

**DOI:** 10.3389/fpls.2026.1958940

**Published:** 2026-09-14

**Authors:** Rong Huang, Xinyi Zhang, Qingrui Meng, Yu Meng, Ling Tang, Siting Lu, Weicheng Wang, Baosheng Shi, Yanhua Pan

**Affiliations:** 1 Institute of Fruit and Floriculture Research, Gansu Academy of Agricultural Sciences, Lanzhou, China; 2 College of Forestry (College of Landscape Architecture and Tourism), Hebei Agricultural University, Baoding, China

**Keywords:** anatomical structure, chlorophyll fluorescence, drought stress, drought trait-integration index, osmotic adjustment, *Paeonia lactiflora*

## Abstract

Drought responses involve coordination across organs and functional levels, yet integrated cultivar strategies remain poorly resolved in herbaceous peony. Five cultivars, ‘Fen Guan’ (FG), ‘Jing Wang Fen’ (JWF), ‘San Dian Hong’ (SDH), ‘Cai Xia’ (CX) and ‘Xiao Ou’ (XO), were exposed for 30 d to nominal target regimes of 65% (CK), 45% (mild drought, LD), 25% (moderate drought, MD) and 5% (severe drought, SD) of substrate maximum water-holding capacity. We quantified leaf water and functional traits, pigment and membrane status, antioxidant enzymes and osmolytes, chlorophyll fluorescence, and leaf and root anatomy. Cultivar differences were evaluated using two-factor models, log response ratios, descriptive principal component analysis, an experiment-specific drought trait-integration index (DTI) and bootstrap resampling. SD reduced relative water content and chlorophyll index, increased relative electrolyte conductivity, and generally induced proline and soluble-sugar accumulation, whereas antioxidant activities, PSII energy fluxes and anatomical responses were cultivar dependent. SDH showed the smallest decline in relative water content and comparatively strong maintenance of PSII photochemical performance. FG showed the largest osmolyte response, but this response did not produce the highest integrated performance. CX showed the largest decline in relative water content and the greatest increase in relative electrolyte conductivity. XO combined pigment-NBI maintenance, photosystem stability and root vascular structural adjustment. Its overall DTI was 0.346 (95% CI, 0.239–0.500). XO ranked first in 99.9% of bootstrap resamples among the five cultivars under the present experimental conditions. These results distinguish stress-response magnitude from functional maintenance and identify XO as the leading candidate for subsequent drought-tolerance validation.

## 1 Introduction

Drought is a major environmental constraint on plant growth and physiological function. Declining soil water availability lowers tissue water potential and turgor, thereby restricting cell expansion, stomatal regulation and photosynthetic carbon assimilation (Lawlor and Cornic, 2002; Chaves et al., 2009; Bashir et al., 2021).When absorbed light energy exceeds metabolic demand, chlorophyll status, photosystem II (PSII) photochemistry and electron transport may change alongside reactive oxygen species accumulation, membrane leakage and lipid peroxidation (Baker, 2008; Stirbet and Govindjee, 2011; Murchie and Lawson, 2013; Kalaji et al., 2014; Shankar and Prasad, 2023). Plants also accumulate osmolytes, adjust antioxidant metabolism and modify leaf and root traits (Poorter et al., 2009; Comas et al., 2013; Pérez-Harguindeguy et al., 2013; Lynch, 2018; Strock and Lynch, 2020; Charng et al., 2023; Renzetti et al., 2024). Because these responses span organs and functional levels, individual response amplitudes do not necessarily indicate preserved function. Comparative assessment therefore benefits from integrating water status, membrane stability, osmolyte and antioxidant responses, PSII photochemistry and organ structure.

Herbaceous peony (*Paeonia lactiflora* Pall.) is an important perennial ornamental and cut-flower crop whose cultivars differ in vigor and environmental response (Li et al., 2020). Water deficit alters leaf and root water status, membrane integrity, osmolyte accumulation and antioxidant activity, with consequences for plant growth (Li et al., 2020). Other Paeonia species also show leaf dehydration, altered photochemical performance and oxidative injury under drought (Zhang et al., 2019; Zhao et al., 2020). As a perennial geophyte, herbaceous peony may depend on both leaf functional maintenance and root structural adjustment. A coordinated assessment across a water gradient can therefore support cultivar comparison while avoiding inference from any single stress marker.

Previous work has identified several drought responses in Paeonia. Li et al. (2020) compared leaf and fibrous-root responses of two herbaceous peony cultivars across soil-water levels and found cultivar-, organ- and severity-dependent changes in water status, electrolyte leakage, malondialdehyde, osmolytes and antioxidant enzymes. Tree peony studies likewise reported changes in hydration, photochemical traits and oxidative status under drought (Zhang et al., 2019; Zhao et al., 2020). Chlorophyll fluorescence can resolve PSII reaction-center status, excitation-energy trapping and electron transport, but its variables describe distinct photochemical processes and are not direct proxies for net CO_2_ assimilation (Baker, 2008; Stirbet and Govindjee, 2011; Murchie and Lawson, 2013; Kalaji et al., 2014). Leaf functional traits describe resource investment, whereas root anatomy describes structural allocation that may be relevant to water acquisition and transport (Poorter et al., 2009; Comas et al., 2013; Pérez-Harguindeguy et al., 2013; Lynch, 2018; Strock and Lynch, 2020). These findings establish a multilayered response, but individual dimensions are often reported separately.

Few studies have integrated leaf water and functional traits, pigment and membrane status, osmolyte and antioxidant responses, PSII energy fluxes, and leaf-root anatomy within one graded-water experiment (Zhang et al., 2019; Li et al., 2020; Zhao et al., 2020). Moreover, the absolute functional state under stress differs conceptually from the magnitude of change relative to control. Large induction may represent protection, but it may also mark greater stress burden. Composite rankings also depend on trait direction, scale, module composition and stress level. The methodological contribution of this study is therefore the *a priori* integration of complementary maintenance modules with bootstrap confidence intervals, rank probabilities and sensitivity analyses, rather than novelty in the individual measurements.

We compared five herbaceous peony cultivars under control, mild, moderate and severe nominal drought regimes. Measurements encompassed leaf water and functional traits, pigment and membrane variables, antioxidant enzymes and osmolytes, basal fluorescence and PSII energy fluxes, and leaf-root anatomy. *A priori* modules, descriptive principal component analysis and an experiment-specific DTI were used to compare relative performance and ranking uncertainty. We expected cultivars to share core drought responses while differing in their principal constraints, and we tested whether coordinated functional maintenance distinguished cultivars more consistently than the largest response of any single defense trait. This framework was designed to characterize drought-response strategies under the present greenhouse conditions and to identify candidates for subsequent validation.

## 2 Materials and methods

### 2.1 Plant material

Five cultivars of *P. lactiflora* were studied: ‘Fen Guan’ (FG), ‘Jing Wang Fen’ (JWF), ‘San Dian Hong’ (SDH), ‘Cai Xia’ (CX) and ‘Xiao Ou’ (XO). All material was supplied by the Institute of Fruit and Floriculture Research, Gansu Academy of Agricultural Sciences The five cultivars were selected from cultivated *P. lactiflora* germplasm maintained by the institute to represent contrasting ornamental phenotypes and growth characteristics and because sufficient uniform plants were available for replicated comparison.

In September 2024, divisions were collected from healthy, pest- and disease-free stock plants during dormancy. Each division retained five to eight well-developed underground buds and 15–20 cm of healthy fleshy primary root; slender, rotten or damaged roots were removed. The divisions were washed, air-dried and stratified in moist sand beneath an approximately 10-cm sand layer that was kept moist, loose and aerated. In February 2025, divisions with plump buds and intact root systems were selected and transplanted individually into pots.

### 2.2 Growth conditions and pot management

The growth substrate was a sterilized 3:1:1 (v/v/v) mixture of peat, vermiculite and perlite. Plastic pots measuring 25 cm in upper diameter and 25 cm in height were each filled with 1.76 kg of air-dried substrate.

The experiment was conducted in a greenhouse at the Gansu Academy of Agricultural Sciences, Lanzhou, Gansu Province, China. Day/night temperatures were 20–28/12–18 °C and relative humidity was 50–70%. All plants received the same irrigation, fertilization and light management before treatment, and flower buds were removed at the visible-bud stage. Once vegetative growth had stabilized, 20 uniform, pest- and disease-free plants were selected per cultivar.

### 2.3 Experimental design, water treatments and leaf sampling

#### 2.3.1 Determination of maximum substrate water-holding capacity

Air-dried substrate was placed in an experimental pot and watered slowly until drainage from the base became continuous. The pot was covered and left for 24 h. Maximum water-holding capacity (MWHC) was determined independently for each experimental pot and was calculated from the mass of the empty pot, air-dried substrate and saturated pot. All masses were recorded in grams:


$$MWHC\left(\%\right)=\frac{M\_sat-M\_pot-M\_dry}{M\_dry}\times 100$$

where Mpot is the empty-pot mass, Mdry is the air-dried substrate mass and Msat is the mass of the pot plus substrate at maximum water-holding capacity. The target mass for a nominal MWHC fraction r was defined as:


M_target,r=M_pot+M_dry+r(M_sat−M_pot−M_dry)

where Mtarget,r is the target total pot mass (g) and r equals 0.65, 0.45, 0.25 or 0.05 for CK, LD, MD or SD, respectively. These expressions define the gravimetric targets and do not constitute independent continuous measurements of substrate water content.

#### 2.3.2 Water gradient and replication

The experiment followed a completely randomized design with four treatments: control (CK), maintained at 60–70% MWHC (target value: 65%); mild drought (LD), 40–50% MWHC (target value: 45%); moderate drought (MD), 20–30% MWHC (target value: 25%); and severe drought (SD), 3–10% MWHC (target value: 5%). Five cultivars were subjected to four drought levels, with each treatment comprising three replicates of three plants each, for a total of 180 plants. The plant/pot was the biological experimental unit. Initial substrate water-content calibration and maximum water-holding capacity (MWHC) were determined prior to the experiment. Substrate moisture was adjusted at 3-day intervals to keep gravimetric water content within the specified target ranges. The gravimetric measurement data are shown in **Supplementary Table 4**. The experimental design is summarized in **Supplementary Figure 1** (Chahal et al., 2018).

#### 2.3.3 Leaf sampling

For leaf sampling, the 3rd to 8th healthy, fully expanded leaves were selected from each plant. Following measurements of chlorophyll fluorescence, chlorophyll index, and nitrogen index, leaves were harvested from each plant. A subsample was retained for leaf trait and electrolyte leakage analyses, while the remaining leaves were pooled, individually wrapped in aluminum foil, immediately flash-frozen in liquid nitrogen, and stored at −80°C for subsequent determination of malondialdehyde (MDA) and other physiological parameters. All measurements were performed in triplicate.

### 2.4 Trait measurements

#### 2.4.1 Leaf morphology and water-related traits

Ten leaves were randomly selected from each treatment and measured. Leaf length (LL), leaf width (LW), leaf area (LA) and leaf perimeter (LC) were measured with a CI-203 leaf-area meter. Mechanical leaf thickness (MLT) was measured with a digital caliper (0.02-mm resolution) at the upper, middle and lower positions of the central lamina while avoiding the main vein. Values were averaged within leaves and then within each plant (Pérez-Harguindeguy et al., 2013).

After morphological measurements, leaf fresh mass (FW) was recorded. Leaves were rehydrated in darkness at 5 °C for 12 h and weighed to obtain saturated fresh mass (SFW). They were then heated at 105 °C for 15 min and dried at 75 °C to constant mass to determine dry mass (DW) (Pérez-Harguindeguy et al., 2013; Tian et al., 2025).

Relative water content (RWC) was calculated as:


$$\text{RWC}\left(\%\right)=\frac{\text{FW}-\text{DW}}{\text{SFW}-\text{DW}}\times 100$$

Specific leaf area (SLA) and leaf dry matter content (LDMC) were calculated as:


$$\text{SLA}=\frac{\text{LA}}{\text{DW}}$$

$$\text{LDMC}=\frac{\text{DW}}{\text{SFW}}$$

#### 2.4.2 Chlorophyll and nitrogen indices

Chlorophyll index and nitrogen balance index (NBI) were measured *in situ* and non-destructively using a DUALEX SCIENTIFIC+ portable polyphenol-chlorophyll meter (Force-A, France). For each treatment, ten mature and healthy functional leaves located in the middle of the canopy were selected for measurement. The main vein was avoided, and the lamina was held flat and centered in the sensor head. Three consecutive readings were taken per leaf. Readings were averaged first within leaves and then across the three leaves of each plant; the plant mean constituted one biological replicate (Goulas et al., 2004; Cartelat et al., 2005).

#### 2.4.3 Relative electrolyte conductivity and malondialdehyde

The harvested leaves were rinsed with deionized water and gently blotted dry with filter paper. Equal tissue masses were immersed in equal volumes of deionized water under standardized extraction conditions before initial conductivity was measured. Tissues were then boiled, cooled to room temperature and measured for total conductivity. A water blank from the same batch provided background correction. Relative electrolyte conductivity (REC) was calculated as follows (Shankar and Prasad, 2023):


$$\text{REC}=\frac{{\text{EC}}_{1}-{\text{EC}}_{0}}{{\text{EC}}_{2}-{\text{EC}}_{0}}$$

Malondialdehyde (MDA) was determined with a microassay kit (MDA-1-Y; Suzhou Comin Biotechnology Co., Ltd., China). Leaf tissue (0.5 g) was homogenized in extraction buffer on ice and centrifuged at 10,000 r min^–1^ for 15 min at 4 °C. The reaction followed the manufacturer’s instructions, and absorbance was measured at 450, 532 and 600 nm. Three biological replicates were analyzed per treatment (Hodges et al., 1999).

#### 2.4.4 Antioxidant-enzyme activities and osmolytes

Leaf tissue (0.5 g), with the main vein removed, was homogenized on ice in 5 mL of pre-chilled 0.05 mol L^–1^ phosphate buffer. The homogenate was centrifuged at 12,000 r min^–1^ for 20 min at 4 °C, and the supernatant was used as the crude enzyme extract.

1. Superoxide dismutase (SOD) activity was determined using NBT-method assay kits (Cat. No. SOD-1-Y, Comin Biotech, Suzhou, China). The reaction system was prepared following the manufacturer’s instructions, and absorbance was read at 450 nm after photochemical reaction. One unit (U) was defined as the enzyme amount inhibiting 50 % of NBT photoreduction. Enzyme activity was normalized to sample fresh weight and expressed as U·g^–1^ FW.
2. Peroxidase (POD) activity was assayed with micro-method kits (Cat. No. POD-1-Y, Comin Biotech, Suzhou, China). Reaction substrates were added to crude enzyme extracts according to kit protocols, and time-dependent absorbance changes were recorded at 470 nm. Activity values were normalized on a fresh-weight basis.
3. Catalase (CAT) activity was measured by ultraviolet-absorption assay kits (Cat. No. CAT-1-Y, Comin Biotech, Suzhou, China). The reaction mixture was prepared as instructed, and time-course absorbance was detected at 510 nm. Enzyme activity was normalized to fresh weight and expressed as U·g^–1^·min^–1^ FW (Giannopolitis and Ries, 1977; Karataş et al., 2014; Tian et al., 2024; Yang et al., 2025).
4. Free proline (Pro) content was determined using micro-assay kits (Cat. No. PRO-1-Y, Comin Biotech, Suzhou, China). Briefly, 0.5 g of leaf tissue was weighed and extracted with the kit-supplied extraction solution in a boiling-water bath for 15 min. After cooling, the mixture was centrifuged and the supernatant was collected. The color-developing reaction was performed following the manufacturer’s instructions, and absorbance was measured at 520 nm.
5. Soluble sugar (SS) content was determined using micro-assay kits (Cat. No. G0501W, Comin Biotech, Suzhou, China) based on the anthrone-sulfuric acid colorimetric method. Leaf samples were homogenized under ice-bath conditions, followed by shaking extraction in a water bath at 50 °C. After centrifugation, the supernatant was mixed with anthrone working solution and concentrated sulfuric acid, then subjected to color development in a 95–100 °C water bath for 10 min. Upon cooling, absorbance was recorded at 620 nm.
6. Soluble protein (SP) content was determined using micro-assay kits (Cat. No. G0417W, Comin Biotech, Suzhou, China) via the Coomassie Brilliant Blue G-250 staining method. Leaves were homogenized on ice and centrifuged to obtain the supernatant. The supernatant was mixed with Coomassie Brilliant Blue G-250 reagent, incubated at 25 °C for 10 min, and absorbance was determined at 600 nm (Bradford, 1976; Zhao et al., 2019; Prysiazhniuk et al., 2023).

#### 2.4.5 Chlorophyll-fluorescence parameters

OJIP transients were recorded at the end of the water treatments using a Handy PEA fluorometer (Hansatech Instruments, UK). Mature leaves were dark-adapted for 30 min and then exposed to saturating red light at 3000 μmol m^-2^ s^–1^. Fluorescence transients were recorded for 1 s and analyzed using the JIP test. All measurements were taken during the same period of the day (Stirbet and Govindjee, 2011; Padhi et al., 2021).

Sixteen parameters were analyzed: Fo, Fm, Fv, Fo/Fm, Fv/Fm, Fv/Fo, ABS/RC, DIo/RC, TRo/RC, ETo/RC, REo/RC, PIabs, DIo/CSo, TRo/CSo, ETo/CSo and REo/CSo. Fo, Fm, Fv and the energy fluxes are reported in instrument or arbitrary units; all ratios and PIabs are dimensionless (Stirbet and Govindjee, 2011; Padhi et al., 2021).

#### 2.4.6 Leaf and root anatomy

At the end of the treatments, three independent plants were sampled per cultivar × drought-treatment combination. Leaf samples were excised from the middle of mature functional leaves while avoiding the main vein; root samples were taken from newly formed roots at comparable developmental stages. Samples were fixed in FAA for 24–48 h, dehydrated, cleared, paraffin embedded and sectioned at 8–10 μm. Sections were stained with safranin-fast green and imaged with a digital slide scanner. Traits were measured in CaseViewer. Five microscopic fields were averaged for each plant before inference. Leaf traits included anatomical leaf thickness, palisade- and spongy-mesophyll thickness, and upper- and lower-epidermis thickness. Root traits included root diameter, periderm thickness, phloem thickness, cambium thickness and xylem-region width (Kosakai, 1973; Ma et al., 1993).

### 2.5 Data processing and statistical analysis

Data were entered in Excel. Data cleaning, trait calculation, statistical analysis and figure preparation were performed in R 4.4.2.

#### 2.5.1 Trait-wise two-factor analysis and multiple comparisons

For each trait, a separate cultivar × water factorial model was fitted:


$$\text{g}({\text{Y}}_{\text{ijk}})=\mu +{\text{C}}_{\text{i}}+{\text{W}}_{\text{j}}+{\left(\text{C}\times \text{W}\right)}_{\text{ij}}+\epsilon_{\text{ijk}}$$

Here, Yijk is the trait value for independent plant k of cultivar i under drought treatment j. Cultivar, drought treatment and their interaction were fixed effects, and the plant was the experimental unit. Trait-specific models were fitted with stats::lm as g(Y) ~ cultivar × water. Type III tests were obtained with car::Anova(type = 3). The function g(·) denotes the prespecified analysis scale selected from diagnostic performance, not statistical significance. Within-plant measurements were averaged before model fitting.

Residual normality was assessed with Shapiro–Wilk tests and Q–Q plots, and homogeneity was assessed with the Brown–Forsythe procedure based on absolute deviations from group medians. Across the 58 analyzed traits, 19 were modelled on the raw scale, 23 after log transformation, 13 after square-root transformation and three after logit transformation. Twenty-five traits underwent aligned-rank-transform sensitivity checks because diagnostic concerns remained; these checks were not substituted selectively for less significant parametric results. Estimated marginal means were calculated with emmeans. Tukey-adjusted comparisons evaluated drought treatments within cultivar and cultivars within treatment, whereas Dunnett-adjusted contrasts compared LD, MD and SD with CK. Benjamini–Hochberg false-discovery-rate control was applied separately across the 58 cultivar, drought-treatment and interaction omnibus P values (Benjamini and Hochberg, 1995; Nanda et al., 2021). Omnibus effects are reported in **Supplementary Table 1** and complete results in **Supplementary Table 2**.

#### 2.5.2 Log response ratios

Model-based log response ratios for each drought treatment relative to the 65% MWHC control of the same cultivar were calculated from trait-wise estimated marginal means. For cultivar c under water regime w, the estimated marginal mean on the log scale was (Hedges et al., 1999):


μ^cwlog=EMM[ln(Yijk)|Ci=c, Wj=w]

where c and w denote cultivar and water regime, respectively. The model-based log response ratio was calculated as:


$$\ln RR_{cw}^{\text{model}}={\hat{\mu }}_{cw}^{\log}-{\hat{\mu }}_{c,65}^{\log}$$

where w ∈ {45, 25, 5}. Percentage change relative to the 65% MWHC control was calculated as:


$$\Delta_{cw}(\%)=[\exp(\ln RR_{cw}^{\text{model}})-1]\times 100$$

Model contrasts provided 95% confidence intervals (CIs) for log response ratios and Dunnett-adjusted P values; these were used for CK-referenced effect plots and trait-wise inference.

For descriptive modules and principal component analysis (PCA), the group mean of trait t for cultivar c under water regime w ( $\bar{Y}\text{cwt}$) was used to calculate the log response ratio:


$$\ln RR_{cwt}^{\text{module}}=\ln(\frac{{\bar{Y}}_{cwt}}{{\bar{Y}}_{c,65,t}})$$

Model-based log response ratios were used for trait-wise inference, whereas log response ratios calculated from group means were used only for module analysis and PCA. For traits containing zero or negative values, no pseudocount was added and ratio-based log response ratios were not calculated (Hedges et al., 1999).

#### 2.5.3 Principal component analysis

Before PCA, eight descriptive modules and their constituent traits were defined *a priori* to limit redundancy and unequal weighting: leaf water/function (RWC, leaf area, SLA and LDMC), pigment/NBI (chlorophyll index and NBI), membrane stability (REC and MDA), antioxidant response (CAT, SOD and POD), osmolyte response (Pro, SP and SS), photosystem stability (Fv/Fm, PIabs, ETo/CSo and REo/CSo), leaf anatomy (anatomical leaf thickness, palisade-to-spongy ratio and epidermal proportion) and root anatomy (root diameter, xylem-region width/root diameter and periderm thickness/root diameter) (Nardo et al., 2005).Trait selection was not based on PCA loadings.

Trait group means for the 20 cultivar × water combinations were Z-standardized across combinations:


$$Z_{cwt}^{\text{raw}}=\frac{{\bar{Y}}_{cwt}-{\bar{Y}}_{\cdot \cdot t}}{{\text{SD}}_{t}^{\text{raw}}}$$

where $\bar{Y}$··t and SDt(raw) are the overall mean and standard deviation, respectively, of trait t across the 20 treatment combinations.

Log response ratios of trait group means for the 15 drought combinations were likewise Z-standardized across combinations:


$$Z_{cwt}^{\ln RR}\;=\;\frac{\ln RR_{cwt}^{\text{module}}-\ln\bar{R}R_{\cdot \cdot t}}{{\text{SD}}_{t}^{\ln RR}}$$

Each module score was the equally weighted mean of its constituent Z scores. REC and MDA scores were multiplied by −1 so that less damage produced a higher membrane-stability score. Other descriptive modules retained their observed direction; anatomical thickening or thinning was not assigned an *a priori* advantage.

Separate centered and scaled PCAs were fitted with stats::prcomp(center = TRUE, scale. = TRUE) to the eight-module matrices for the 20 raw combinations and 15 drought lnRR combinations. PCA was used only to summarize multivariate covariation. It was not used for inferential testing, causal interpretation, trait selection or DTI weighting (Jolliffe and Cadima, 2016).

#### 2.5.4 Four-module drought trait-integration index

The DTI was specified *a priori* as an experiment-specific relative integration index for the five cultivars and conditions studied here. It combined four functional-maintenance domains: resource/water maintenance, membrane stability, photosystem stability and leaf-root structural stability. At each drought level, constituent traits and completed modules were standardized across the five cultivars. Equal module weighting was used because no independent training dataset or sufficient prior evidence supported differential weights, and equal weighting prevented modules with more measured traits from dominating the index. The calculations were as follows:


$$Z_{w}\left(X_{cw}\right)=\frac{X_{cw}-{\bar{X}}_{\cdot w}}{SD\left(X_{\cdot w}\right)}$$

where X_cw_ is the group mean for cultivar c at drought level w, and ${\bar{X}}_{\cdot w}$ and $\text{SD}\left({\text{X}}_{\cdot \text{w}}\right)$ are the mean and standard deviation, respectively, across the five cultivars at that drought level. This standardization expresses each module as relative performance among cultivars within the same drought level.

The resource and water-maintenance module comprised RWC, chlorophyll index and NBI:


$${\text{R}}_{\text{water,cw}}={\text{Z}}_{\text{w}}\left({\text{RWC}}_{\text{cw}}\right)$$

$${\text{R}}_{\text{pigment,cw}}=\frac{{\text{Z}}_{\text{w}}\left({\text{Chl}}_{\text{cw}}\right)+{\text{Z}}_{\text{w}}\left({\text{NBI}}_{\text{cw}}\right)}{2}$$

$${\text{R}}_{\text{cw}}={\text{Z}}_{\text{w}}\left(\frac{{\text{R}}_{\text{water,cw}}+{\text{R}}_{\text{pigment,cw}}}{2}\right)$$

where Chl and NBI denote chlorophyll index and nitrogen balance index, respectively.

REC and MDA were reverse-scored in the membrane-stability module, such that lower membrane damage produced a higher module score:


$${\text{M}}_{\text{cw}}={\text{Z}}_{\text{w}}\left[\frac{-{\text{Z}}_{\text{w}}\left({\text{REC}}_{\text{cw}}\right)-{\text{Z}}_{\text{w}}\left({\text{MDA}}_{\text{cw}}\right)}{2}\right]$$

The photosystem-stability module comprised Fv/Fm, PIabs, ETo/CSo and REo/CSo:


$${\text{P}}_{\text{cw}}={\text{Z}}_{\text{w}}\left[\frac{{\text{Z}}_{\text{w}}\left({\left({\text{F}}_{\text{v}}/{\text{F}}_{\text{m}}\right)}_{\text{cw}}\right)+{\text{Z}}_{\text{w}}\left({\text{PIabs}}_{\text{cw}}\right)+{\text{Z}}_{\text{w}}\left({\left({\text{ET}}_{\text{o}}/{\text{CS}}_{\text{o}}\right)}_{\text{cw}}\right)+{\text{Z}}_{\text{w}}\left({\left({\text{RE}}_{\text{o}}/{\text{CS}}_{\text{o}}\right)}_{\text{cw}}\right)}{4}\right]$$

The structural module quantified the retention of each anatomical trait relative to CK in the same cultivar. For structural trait *t*, the stability score was calculated as:


$${\text{Stability}}_{cwt}=-\left|\ln\left(\frac{{\bar{Y}}_{cwt}}{{\bar{Y}}_{c,\text{CK,}t}}\right)\right|$$

where ${\bar{Y}}_{cwt}$ and ${\bar{Y}}_{c,\text{CK},t}$ are the group means for cultivar *c* at drought level *w* and CK, respectively, for trait *t*. Values closer to zero indicate a smaller change from CK. This definition does not assume that either anatomical thickening or thinning is intrinsically advantageous.

The leaf structural submodule comprised anatomical leaf thickness, the palisade-to-spongy ratio and epidermal proportion; the root structural submodule comprised root diameter, the xylem-region width/root-diameter ratio and the periderm-thickness/root-diameter ratio:


$${\text{A}}_{\text{leaf,cw}}=\frac{{\text{Z}}_{\text{w}}\left({\text{Stability}}_{\text{cw,LT}}\right)+{\text{Z}}_{\text{w}}\left({\text{Stability}}_{\text{cw,PSR}}\right)+{\text{Z}}_{\text{w}}\left({\text{Stability}}_{\text{cw,ER}}\right)}{3}$$

$${\text{A}}_{\text{root,cw}}=\frac{{\text{Z}}_{\text{w}}\left({\text{Stability}}_{\text{cw,RD}}\right)+{\text{Z}}_{\text{w}}\left({\text{Stability}}_{\text{cw,XR}}\right)+{\text{Z}}_{\text{w}}\left({\text{Stability}}_{\text{cw,PR}}\right)}{3}$$

$${\text{A}}_{\text{cw}}={\text{Z}}_{\text{w}}\left(\frac{{\text{A}}_{\text{leaf,cw}}+{\text{A}}_{\text{root,cw}}}{2}\right)$$

LT, PSR and ER denote leaf thickness, palisade-to-spongy ratio and epidermal proportion, respectively; RD, XR and PR denote root diameter, xylem-region width/root-diameter ratio and periderm-thickness/root-diameter ratio, respectively. The leaf and root submodules each contributed 50%.

The four-module DTI was calculated as:


$${\text{DTI}}_{\text{cw}}=\frac{{\text{R}}_{\text{cw}}+{\text{M}}_{\text{cw}}+{\text{P}}_{\text{cw}}+{\text{A}}_{\text{cw}}}{4}$$

Overall DTI for each cultivar was the mean DTI across LD, MD and SD:


$${\text{Overall DTI}}_{\text{c}}=\frac{{\text{DTI}}_{\text{c,LD}}+{\text{DTI}}_{\text{c,MD}}+{\text{DTI}}_{\text{c,SD}}}{3}$$

Relative performance under severe drought was defined as:


$${\text{S}}_{\text{c}}={\text{DTI}}_{\text{c,SD}}$$

Consistency of relative performance across the water gradient was defined as:


$${\text{CRC}}_{\text{c}}=-\text{SD}\left({\text{DTI}}_{\text{c,LD}}{\text{,DTI}}_{\text{c,MD}}{\text{,DTI}}_{\text{c,SD}}\right)$$

Values of CRC closer to zero indicate a more consistent relative position across LD, MD and SD, not stability across environments or absolute physiological stability. Antioxidant-response and osmolyte-response modules were used only to characterize stress-response strategies. They were excluded from DTI weighting because stronger induction may represent protection or greater stress burden and cannot automatically be scored as a tolerance advantage.

DTI represents the relative performance of these five cultivars within the present 30-d greenhouse pot experiment, fixed substrate and measured trait framework. It is neither an absolute drought-tolerance threshold nor evidence of environment-independent field adaptation.

#### 2.5.5 Bootstrap resampling and sensitivity analysis

Uncertainty in DTI and cultivar rankings was evaluated by nonparametric bootstrap resampling. Independent plants were resampled with replacement 2,000 times within each cultivar × drought-treatment × trait combination. Group means, lnRR values, Z scores, module scores, DTI values and ranks were recalculated in every iteration. Plants were also the resampling unit for anatomy; microscopic fields from one plant were not treated as independent observations.

Percentile 95% CIs were calculated for overall DTI, performance under SD, cross-gradient consistency and pairwise DTI differences. Rank probabilities were tabulated after cultivars were reranked within every bootstrap iteration. This bootstrap quantifies sampling uncertainty conditional on the biological replicates and design of this experiment. It does not quantify uncertainty across environments, years, substrates, developmental stages or field conditions (Rosenblad, 2009).

Four sensitivity schemes were evaluated. Scheme A was the primary four-module formulation; whereas scheme B omitted the structural module:


$${\text{DTI}}_{\text{cw}}^{\text{B}}=\frac{{\text{R}}_{\text{cw}}+{\text{M}}_{\text{cw}}+{\text{P}}_{\text{cw}}}{3}$$

Scheme C retained four modules but represented membrane stability using REC alone:


$${\text{M}}_{\text{cw}}^{\text{REC}}={\text{Z}}_{\text{w}}\left[-{\text{Z}}_{\text{w}}\left({\text{REC}}_{\text{cw}}\right)\right]$$

Scheme D used an alternative set of structural traits. Leaf traits comprised leaf thickness, palisade-mesophyll thickness and spongy-mesophyll thickness; root traits comprised root diameter, xylem-region width and periderm thickness. Structural stability remained defined as −|ln *RR*| to quantify retention relative to CK, with the leaf and root submodules each contributing 50%.

Spearman rank correlations compared rankings from each sensitivity scheme with the primary scheme, and CIs were estimated by bootstrap resampling. If rankings changed appreciably, the result was considered sensitive to module or representative-trait selection, and an uncertainty-free complete ranking was not reported (Rosenblad, 2009; Chairani, 2021).

## 3 Results

### 3.1 Leaf water status, morphology and membrane-associated traits

None of the three drought treatments significantly affected leaf area, specific leaf area (SLA) or leaf dry matter content (LDMC) in any cultivar relative to CK (Dunnett-adjusted *P* ≥ 0.05) (**Figures 1D, E**). Comparisons among cultivars showed that JWF had a larger leaf area and higher LDMC under LD and SD, whereas SLA did not differ significantly among cultivars under any water regime (**Figure 1A**).

**Figure 1:**
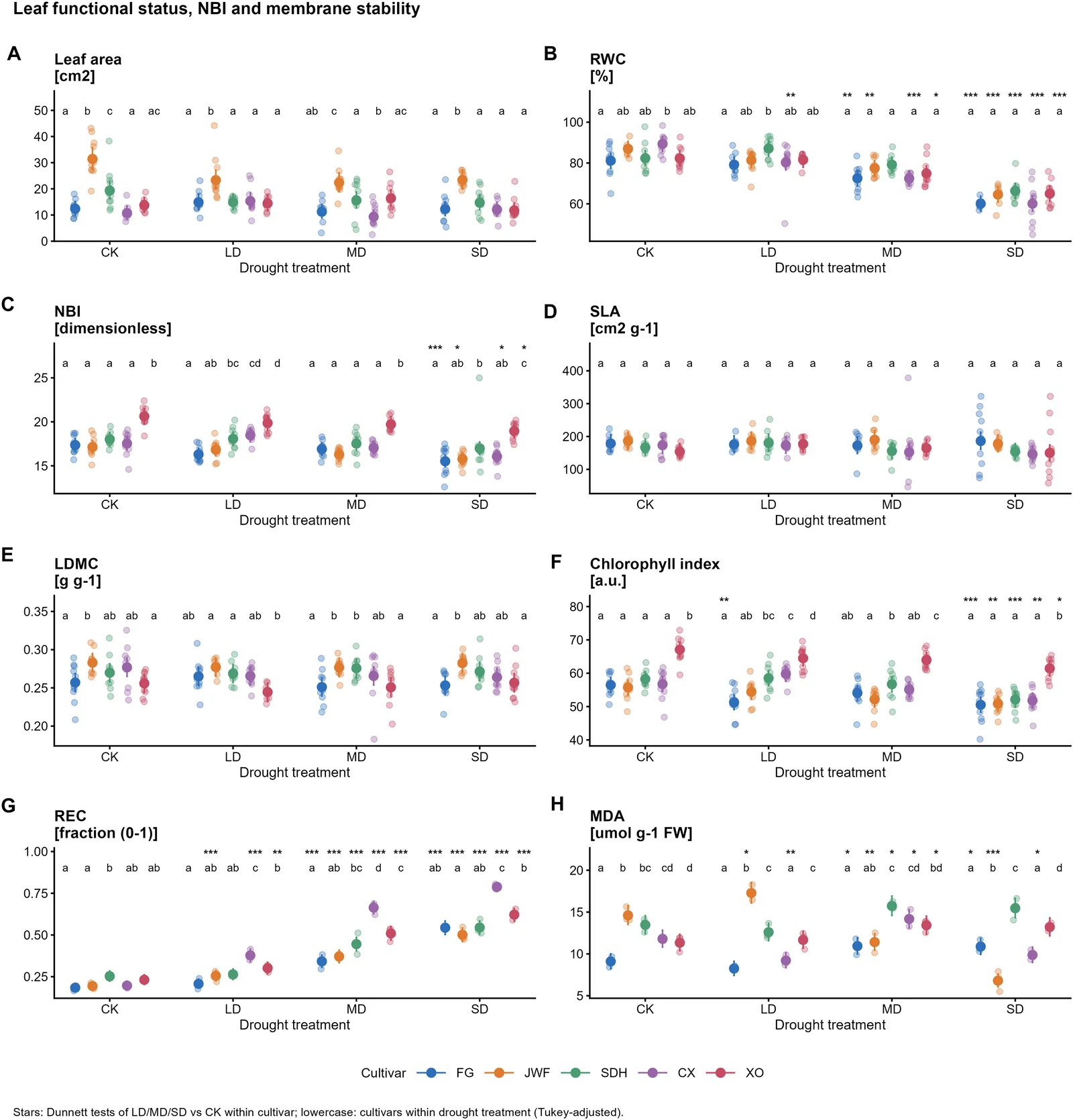
Leaf functional status, nitrogen balance index and membrane stability under graded drought. Cultivars are FG, Fen Guan; JWF, Jing Wang Fen; SDH, San Dian Hong; CX, Cai Xia; and XO, Xiao Ou. Drought treatments are CK, control (65% MWHC); LD, mild drought (45%); MD, moderate drought (25%); and SD, severe drought (5%). Panels show **(A)** leaf area, **(B)** relative water content (RWC), **(C)** nitrogen balance index (NBI), **(D)** specific leaf area (SLA), **(E)** leaf dry matter content (LDMC), **(F)** chlorophyll index, **(G)** relative electrolyte conductivity (REC) and **(H)** malondialdehyde (MDA). Biological n = 3 independent replicates per cultivar × treatment combination, with three plants contributing to each replicate. Plant-level measurements were averaged within each biological replicate to generate one replicate-level value before statistical analysis; within-plant leaf and instrument subsamples were averaged before replicate-level averaging. Faint points represent replicate-level observations. Solid symbols and error bars are model-estimated marginal means and 95% confidence intervals (CIs) from trait-specific cultivar × drought-treatment models. Stars indicate Dunnett-adjusted comparisons of LD, MD, and SD with CK within each cultivar (P< 0.05, P< 0.01, and P< 0.001). Lowercase letters compare cultivars within a treatment; groups sharing a letter do not differ significantly (Tukey-adjusted P ≥ 0.05). NBI is the dimensionless DUALEX nitrogen balance index, not total leaf nitrogen. REC is shown as a fraction from 0 to 1, and a.u. denotes instrument-reported arbitrary units. * indicates P < 0.05, ** indicates P < 0.01, and *** indicates P < 0.001.

RWC declined with increasing drought severity (**Figure 1B**). Under LD, only CX showed a significant decline (11.0%). Under MD, RWC declined by 9.1–18.9% in all cultivars except SDH, whereas SD significantly reduced RWC by 19.4–33.5% in all cultivars, with the largest reduction in CX. Neither LD nor MD significantly affected NBI (**Figure 1C**). Under SD, NBI decreased significantly by 8.0–10.6% in every cultivar except SDH. XO maintained a comparatively high NBI under all three drought treatments and had significantly higher values than the other cultivars under MD and SD. Chlorophyll index decreased significantly only in FG under LD (9.3%), was unchanged under MD, and declined significantly by 8.4–10.9% in all cultivars under SD (**Figure 1F**). XO had a significantly higher chlorophyll index than the other cultivars under each drought treatment.

REC was highly responsive to drought (**Figure 1G**). It increased significantly under LD in JWF, CX and XO and rose by 75.4–237.1% and 115.2–300.9% in all cultivars under MD and SD, respectively. CX had significantly higher REC than the other cultivars under all three drought treatments. MDA responses depended strongly on both cultivar and treatment (**Figure 1H**). Under LD, MDA increased significantly in JWF but decreased in CX. Under MD, it increased significantly in all cultivars except JWF, in which it declined. Under SD, MDA increased significantly in FG, decreased in JWF and CX, and was unchanged in SDH and XO.

### 3.2 Antioxidant-enzyme and osmolyte responses relative to CK

Antioxidant-enzyme activities differed substantially among cultivars and treatments. Under LD, CAT activity decreased by 13.2% in FG and SOD activity increased by 36.9% in CX. POD activity declined by 19.4% in FG and 32.8% in CX but increased by 142.1% in SDH. Under MD, CAT activity did not change significantly in any cultivar; SOD activity increased by 13.6%, 13.1% and 36.9% in FG, SDH and CX, respectively, whereas POD activity decreased by 36.1% in FG and 47.8% in CX and increased by 29.6% in JWF. Under SD, CAT increased only in CX (16.8%) and SOD only in SDH (18.6%); POD declined by 61.7%, 40.1% and 50.9% in FG, SDH and CX, respectively.

Osmolyte responses varied with drought severity and cultivar. Under LD, Pro increased by 102.1%, 17.7% and 66.6% in FG, JWF and XO, respectively, but decreased by 24.9% in SDH. SP increased only in FG (13.1%), whereas SS increased only in FG (28.2%) and SDH (17.0%). Under MD, Pro increased by 667.8%, 48.3%, 37.1% and 89.6% in FG, SDH, CX and XO, respectively, while SP remained unchanged in all cultivars. SS increased significantly in all five cultivars, by 50.4%, 32.1%, 38.1%, 46.9% and 72.7% in FG, JWF, SDH, CX and XO, respectively.

Under SD, all five cultivars accumulated significantly more Pro and SS. Pro increased by 457.2%, 130.4%, 21.2%, 101.3% and 294.3% in FG, JWF, SDH, CX and XO, respectively; the corresponding increases in SS were 120.9%, 43.0%, 78.6%, 83.6% and 109.2%. SP increased only in FG (13.6%) and did not change significantly in the other cultivars (**Figure 2**).

**Figure 2:**
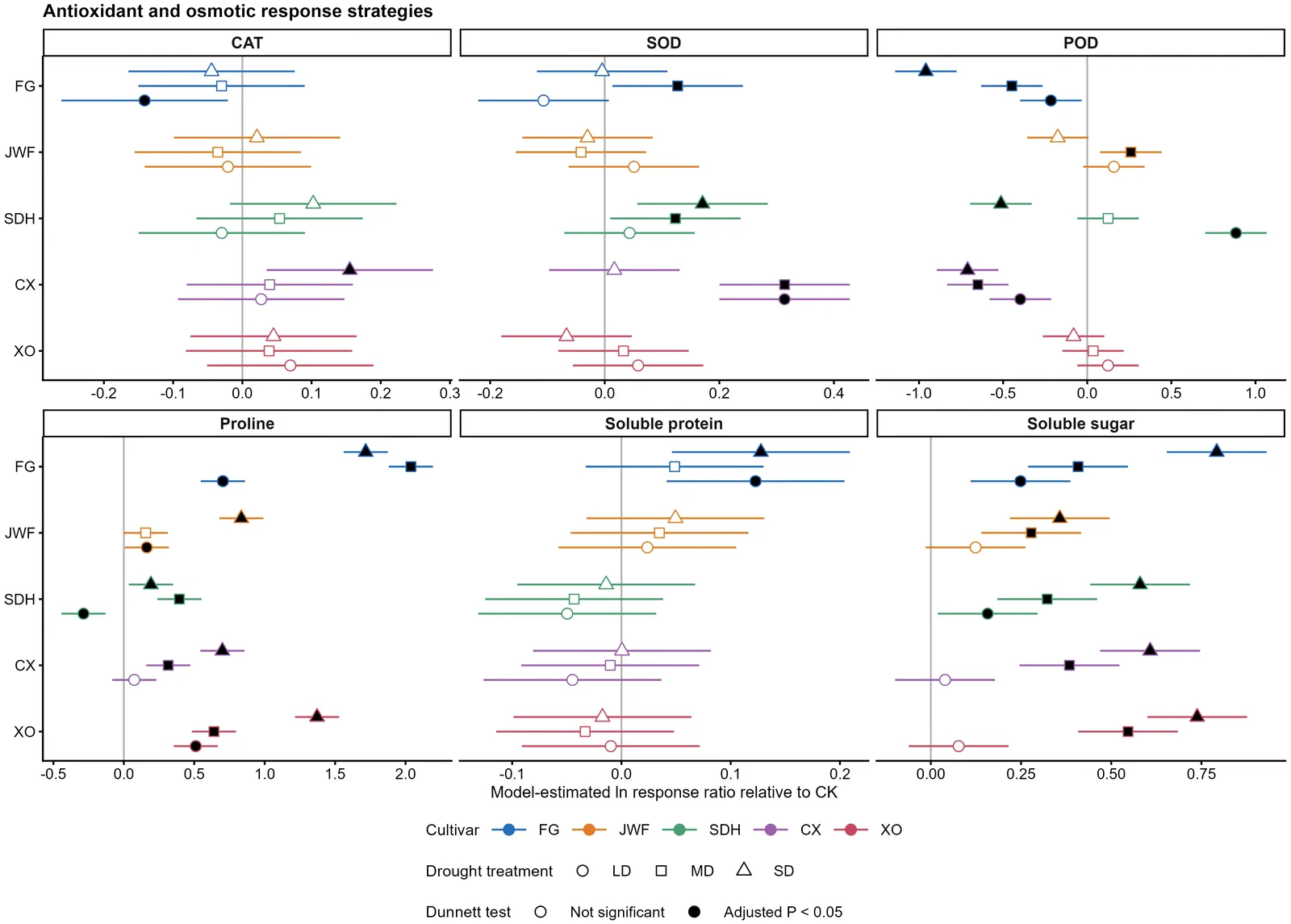
Model-estimated antioxidant and osmolyte responses relative to CK. Cultivars are FG, Fen Guan; JWF, Jing Wang Fen; SDH, San Dian Hong; CX, Cai Xia; and XO, Xiao Ou. Treatments are CK, control (65% MWHC); LD, mild drought (45%); MD, moderate drought (25%); and SD, severe drought (5%). Traits are catalase (CAT), superoxide dismutase (SOD), peroxidase (POD), proline (Pro), soluble protein (SP) and soluble sugars (SS). Biological n = 3 independent replicates per cultivar × treatment combination, with three plants contributing to each replicate. For biochemical assays, tissue from the three plants within each biological replicate was pooled to constitute one biological sample. Technical assay replicates were averaged before statistical analysis and were not treated as independent biological replicates. Points are model-estimated log response ratios (lnRR) for drought treatments relative to CK, and horizontal bars are 95% CIs. The zero line indicates no change relative to CK. Filled symbols indicate Dunnett-adjusted P< 0.05 and open symbols indicate P ≥ 0.05. Symbol shape denotes LD, MD, or SD, and colour denotes cultivar These variables describe antioxidant and osmolyte responses, not direct measurements of osmotic potential.

### 3.3 Basal fluorescence and photosystem energy fluxes

The Z-score heatmap of basal chlorophyll-fluorescence parameters shows the relative levels of Fo, Fm, Fv, Fo/Fm, Fv/Fm and Fv/Fo across cultivars and water regimes (**Figure 3**). LD did not significantly affect any of these variables, and under MD only Fm in FG increased significantly (9.2%). Under SD, Fo increased by 15.6% in CX, Fm and Fv by 12.0% and 11.5%, respectively, in JWF, and Fv/Fm by 4.4% in SDH (Dunnett-adjusted P< 0.05); all other responses were non-significant.

**Figure 3:**
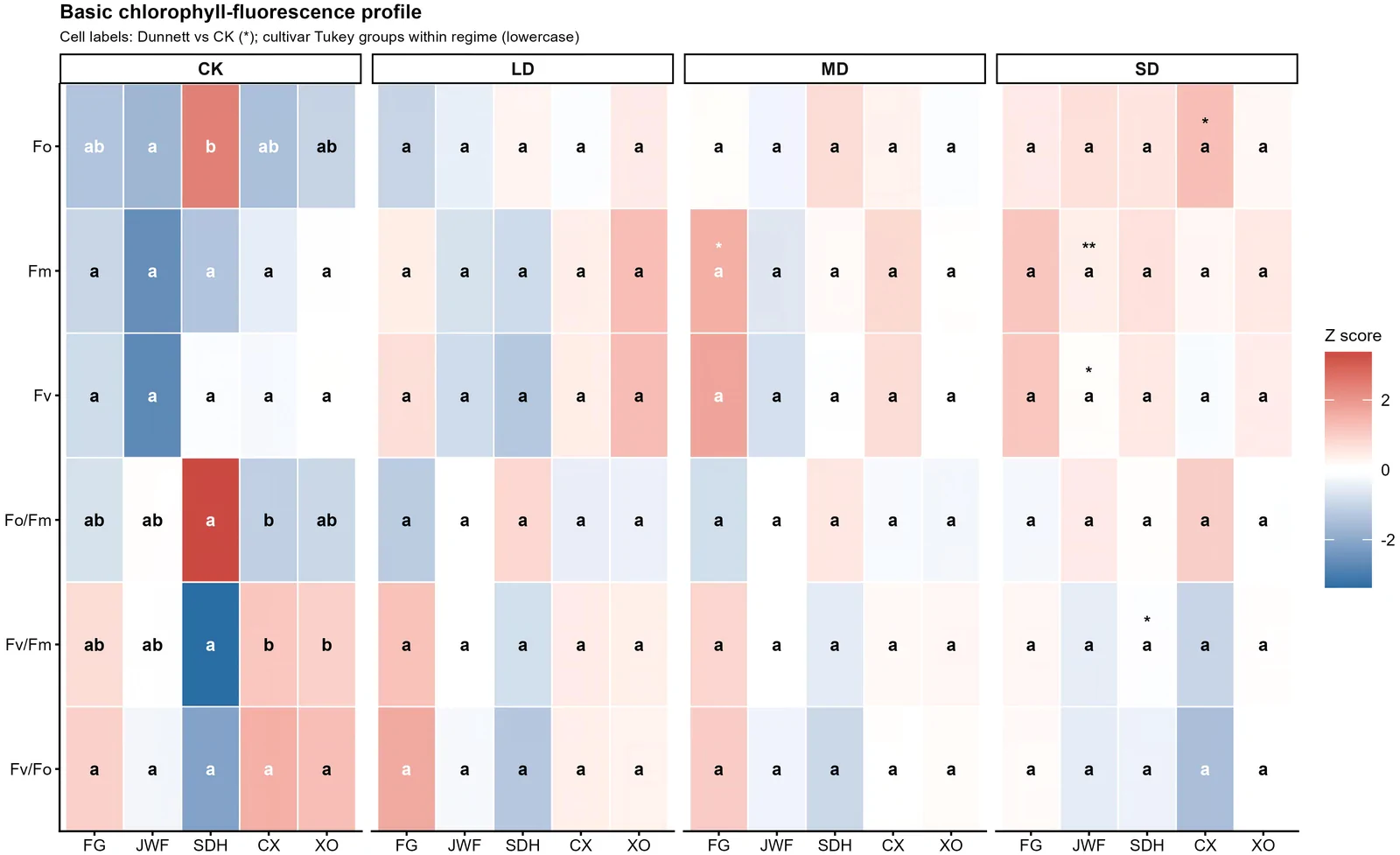
Basal chlorophyll-fluorescence profiles under graded drought. Cultivars are FG, Fen Guan; JWF, Jing Wang Fen; SDH, San Dian Hong; CX, Cai Xia; and XO, Xiao Ou. Treatments are CK, control (65% MWHC); LD, mild drought (45%); MD, moderate drought (25%); and SD, severe drought (5%). Traits are minimum fluorescence (Fo), maximum fluorescence (Fm), variable fluorescence (Fv), Fo/Fm, maximum quantum yield of PSII photochemistry (Fv/Fm) and Fv/Fo. Biological n = 3 independent replicates per cultivar × treatment combination, with three plants measured within each replicate. Plant-level fluorescence measurements were averaged within each biological replicate to generate one replicate-level value before statistical analysis. For each trait, the model-estimated means of the 20 cultivar × treatment combinations were Z-standardized across combinations as Z = (x − μ)/σ, where μ and σ are the mean and standard deviation, respectively, of the 20 model-estimated means for that trait. Colour therefore indicates the relative position of each cultivar × treatment combination within a trait rather than the original measurement unit. Each cell contains compact Tukey group letters: uppercase letters compare treatments within a cultivar, whereas lowercase letters compare cultivars within a treatment. Groups sharing a letter do not differ significantly (Tukey-adjusted P ≥ 0.05). Fluorescence variables describe PSII photochemical performance rather than net CO_2_ assimilation.

At the reaction-center scale, neither LD nor MD significantly affected ABS/RC, DIo/RC, TRo/RC, ETo/RC, REo/RC or PIabs (**Figure 4**). Under SD, only ETo/RC in SDH increased significantly (24.1%), whereas REo/RC in CX declined significantly (23.3%; Dunnett-adjusted P< 0.05). PIabs did not change significantly in any cultivar under SD; the 30.1% reduction in CX approached, but did not reach, significance (P = 0.0562).

**Figure 4:**
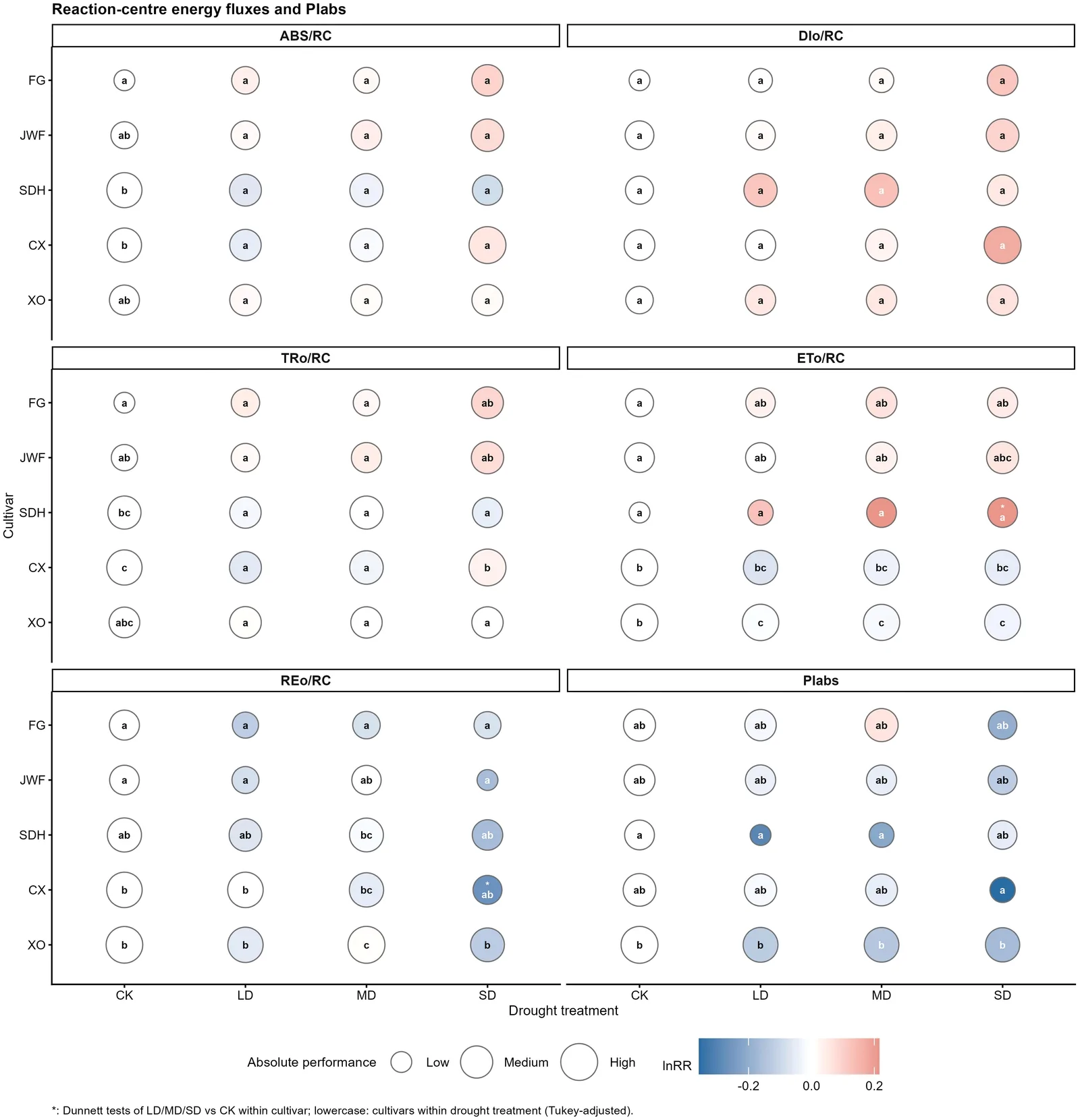
Reaction-center energy fluxes and PIabs under graded drought. Cultivars are FG, Fen Guan; JWF, Jing Wang Fen; SDH, San Dian Hong; CX, Cai Xia; and XO, Xiao Ou. Treatments are CK, control (65% MWHC); LD, mild drought (45%); MD, moderate drought (25%); and SD, severe drought (5%). Panels show absorption (ABS/RC), dissipation (DIo/RC), trapping (TRo/RC), electron transport (ETo/RC) and reduction of PSI end acceptors (REo/RC) per active PSII reaction center, plus the performance index on an absorption basis (PIabs). Biological n = 3 independent replicates per cultivar × treatment combination, with three plants measured within each replicate. Plant-level fluorescence measurements were averaged within each biological replicate to generate one replicate-level value before statistical analysis. Fill colour represents the model-estimated lnRR relative to CK, whereas circle area represents the standardized absolute model-estimated marginal mean. Grey outlines are neutral and do not encode statistical significance. Within bubbles, stars indicate Dunnett-adjusted comparisons with CK, and lowercase letters compare cultivars within a treatment using Tukey-adjusted tests. Groups sharing a letter do not differ (Tukey-adjusted *P* ≥ 0.05); *P < 0.05. By definition, CK lnRR = 0. These fluxes describe PSII photochemical energy allocation.

At the scale of the excited leaf cross-section, LD did not significantly affect DIo/CSo, TRo/CSo, ETo/CSo or REo/CSo (**Figure 5**). Under MD, only ETo/CSo in SDH increased significantly (24.4%). Under SD, TRo/CSo increased by 13.0% in JWF and 12.8% in CX, and ETo/CSo increased by 30.5% in SDH (Dunnett-adjusted P< 0.05); all other energy-flux responses were non-significant.

**Figure 5:**
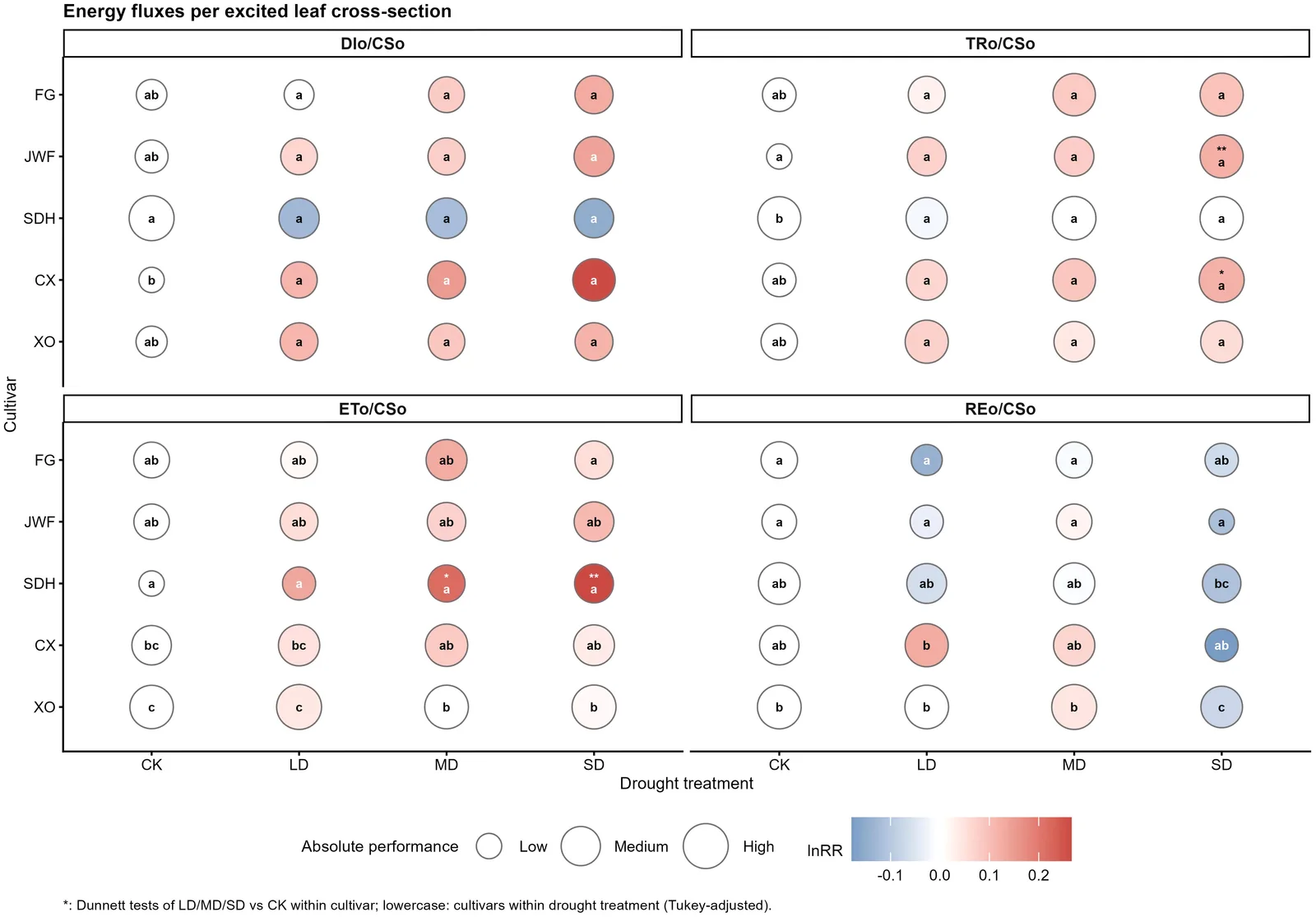
Energy fluxes per excited leaf cross-section under graded drought. Cultivars are FG, Fen Guan; JWF, Jing Wang Fen; SDH, San Dian Hong; CX, Cai Xia; and XO, Xiao Ou. Treatments are CK, control (65% MWHC); LD, mild drought (45%); MD, moderate drought (25%); and SD, severe drought (5%). Panels show energy dissipation (DIo/CSo), trapping (TRo/CSo), electron transport (ETo/CSo) and reduction of PSI end acceptors (REo/CSo) per excited leaf cross-section. Biological n = 3 independent replicates per cultivar × treatment combination, with three plants measured within each replicate. Plant-level fluorescence measurements were averaged within each biological replicate to generate one replicate-level value before statistical analysis. Grey outlines are neutral. Within bubbles, stars show Dunnett-adjusted comparisons with CK and lowercase letters compare cultivars within treatment by Tukey tests. Groups sharing a letter do not differ (Tukey-adjusted P ≥ 0.05); *P< 0.05, **P< 0.01. By definition, CK lnRR = 0. These fluxes describe PSII photochemical energy allocation.

### 3.4 Leaf and root anatomy

Leaf anatomical traits showed pronounced cultivar-specific responses to water regime (**Figure 6A**). Under LD, leaf thickness increased significantly by 11.2% in FG and 12.8% in JWF but decreased by 31.1% in SDH. Palisade-mesophyll proportion declined by 25.8% in FG, whereas palisade- and spongy-mesophyll proportions increased by 34.8% and 41.3%, respectively, in CX. The palisade-to-spongy ratio decreased by 35.3% in FG and 24.4% in JWF, and epidermal proportion increased by 28.7% in SDH. Under MD, leaf thickness increased by 27.1% in CX but declined by 11.3%, 40.1% and 27.1% in JWF, SDH and XO, respectively. Palisade-mesophyll proportion decreased by 25.5% in JWF and 17.9% in CX; spongy-mesophyll proportion decreased by 18.1% in CX; and the palisade-to-spongy ratio decreased by 32.0% in JWF. Epidermal proportion increased by 47.3% in SDH and 29.5% in XO. Under SD, leaf thickness decreased significantly by 29.9%, 24.6% and 20.2% in FG, JWF and SDH, respectively. It increased by 7.3% in CX and decreased by 8.3% in XO, although neither change was significant. Palisade-mesophyll proportion increased by 22.7% in SDH, and epidermal proportion increased by 34.2% in FG and 37.6% in JWF. Spongy-mesophyll proportion and the palisade-to-spongy ratio did not differ significantly between SD and CK in any cultivar (**Figure 6B**).

**Figure 6:**
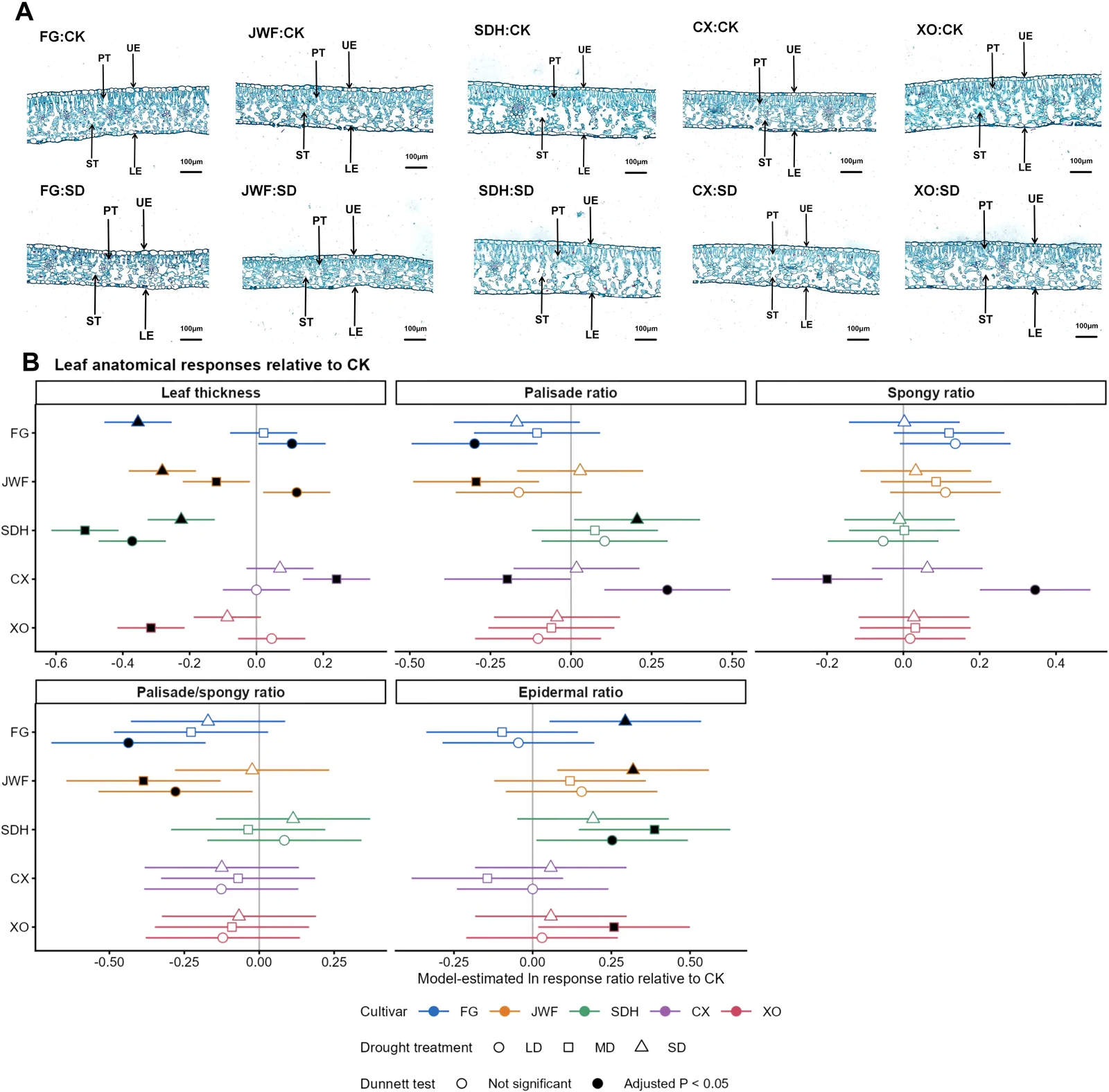
Leaf anatomy under control and severe drought, with quantitative responses referenced to CK. Cultivars are FG, Fen Guan; JWF, Jing Wang Fen; SDH, San Dian Hong; CX, Cai Xia; and XO, Xiao Ou. Treatments are CK, control (65% MWHC); LD, mild drought (45%); MD, moderate drought (25%); and SD, severe drought (5%). **(A)** Representative transverse leaf cross -sections for CK and SD. Labeled structures: UE, upper epidermis; PT, palisade tissue; ST, spongy tissue; LE, lower epidermis. Images retain the supplied calibration (100μm scale bar) and were not selectively enhanced. **(B)** Model-estimated lnRR values for LD, MD and SD relative to CK are shown with 95% CIs. The zero line indicates no change. Circles, squares and triangles denote LD, MD and SD; filled symbols indicate Dunnett-adjusted P< 0.05 and open symbols P ≥ 0.05. Each group contained n = 3 independent plants, and each plant value was the mean of five microscopic fields. Lines are CIs and do not imply paired observations. Absolute dimensions were recorded in μm and ratios are dimensionless.

Root anatomical traits also differed in the direction of their responses across the water gradient (**Figure 7A**). Under LD, root diameter increased by 7.0%, 5.6% and 57.9% in FG, CX and XO, respectively, but decreased by 8.1% in JWF. Periderm thickness declined by 26.1% in XO. Phloem thickness increased by 15.4% in CX and 51.7% in XO. Cambium thickness decreased by 24.9% in JWF but increased by 54.7% in CX. Xylem-region width decreased by 38.3% in JWF and 20.4% in SDH but increased by 75.3% in XO; the xylem proportion decreased by 33.0% in JWF and 20.4% in SDH.

**Figure 7:**
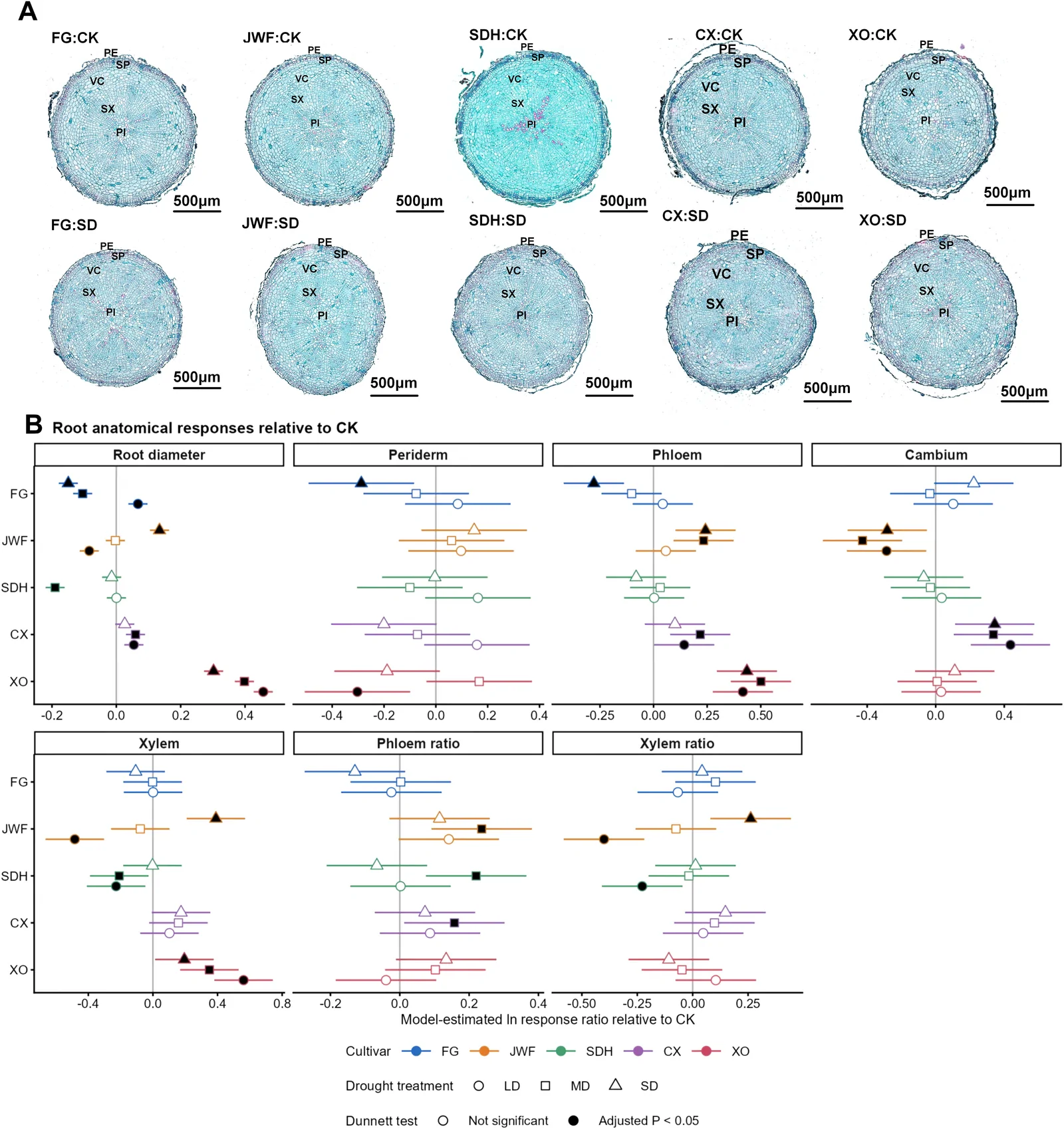
Root anatomy under control and severe drought, with quantitative responses referenced to CK. Cultivars are FG, Fen Guan; JWF, Jing Wang Fen; SDH, San Dian Hong; CX, Cai Xia; and XO, Xiao Ou. Treatments are CK, control (65% MWHC); LD, mild drought (45%); MD, moderate drought (25%); and SD, severe drought (5%). **(A)** Representative transverse root cross - section for CK and SD.Labeled structure: PE,periderm; SP, secondary phloem; VC, vascular cambium; SX, secondary xylem; PI, pith. Images retain the supplied calibration (500μm scale bar) and were not selectively enhanced. **(B)** Model-estimated lnRR values for LD, MD and SD relative to CK are shown with 95% CIs. The zero line indicates no change. Circles, squares and triangles denote LD, MD and SD; filled symbols indicate Dunnett-adjusted *P* < 0.05 and open symbols *P* ≥ 0.05. Each group contained n = 3 independent plants, and each plant value was the mean of five microscopic fields. Lines are CIs and do not imply paired observations. Absolute dimensions were recorded in μm and ratios are dimensionless. Anatomical dimensions indicate structural allocation and do not directly measure hydraulic conductance.

Under MD, root diameter increased by 6.2% in CX and 48.9% in XO but decreased by 9.9% in FG and 17.3% in SDH. Phloem thickness increased by 26.3%, 24.3% and 65.0% in JWF, CX and XO, respectively. Cambium thickness decreased by 34.8% in JWF but increased by 40.1% in CX; xylem-region width decreased by 18.8% in SDH but increased by 41.9% in XO. Phloem proportion also increased by 26.7%, 24.7% and 17.1% in JWF, SDH and CX, respectively, whereas xylem proportion did not differ significantly between MD and CK in any cultivar.

Under SD, root diameter decreased significantly by 13.9% in FG but increased significantly by 14.4% in JWF and 35.3% in XO; changes in SDH and CX were non-significant. Periderm and phloem thickness in FG declined by 25.0% and 24.3%, respectively, whereas phloem thickness increased by 27.4% in JWF and 54.6% in XO. Cambium thickness decreased by 24.6% in JWF but increased by 41.1% in CX. Xylem-region width increased by 47.7% in JWF and 21.5% in XO. Among proportional traits, xylem proportion increased by 30.0% in JWF, whereas phloem proportion did not differ significantly between SD and CK in any cultivar (**Figure 7B**).

### 3.5 Module responses, principal component analysis and DTI

Under LD, standardized module lnRR scores ranged from −1.236 for the antioxidant-response module in FG to 2.330 for the pigment/NBI module in CX. Under MD, scores ranged from −1.041 for membrane stability in CX to 0.991 for osmolyte response in FG. Under SD, they ranged from −1.545 for photosystem stability in CX to 1.844 for osmolyte response in FG (**Figure 8A**). Under SD, the highest and lowest cultivar scores, respectively, were 0.079 (FG) and −1.144 (CX) for leaf water/function; −0.683 (SDH) and −1.425 (FG) for pigment/NBI; 1.095 (JWF) and −0.862 (FG) for membrane stability; 0.435 (SDH) and −1.039 (FG) for antioxidant response; 1.844 (FG) and −0.100 (SDH) for osmolyte response; 0.995 (SDH) and −1.545 (CX) for photosystem stability; 0.487 (SDH) and −0.088 (FG) for leaf anatomy; and 0.822 (JWF) and −0.340 (FG) for root anatomy.

**Figure 8:**
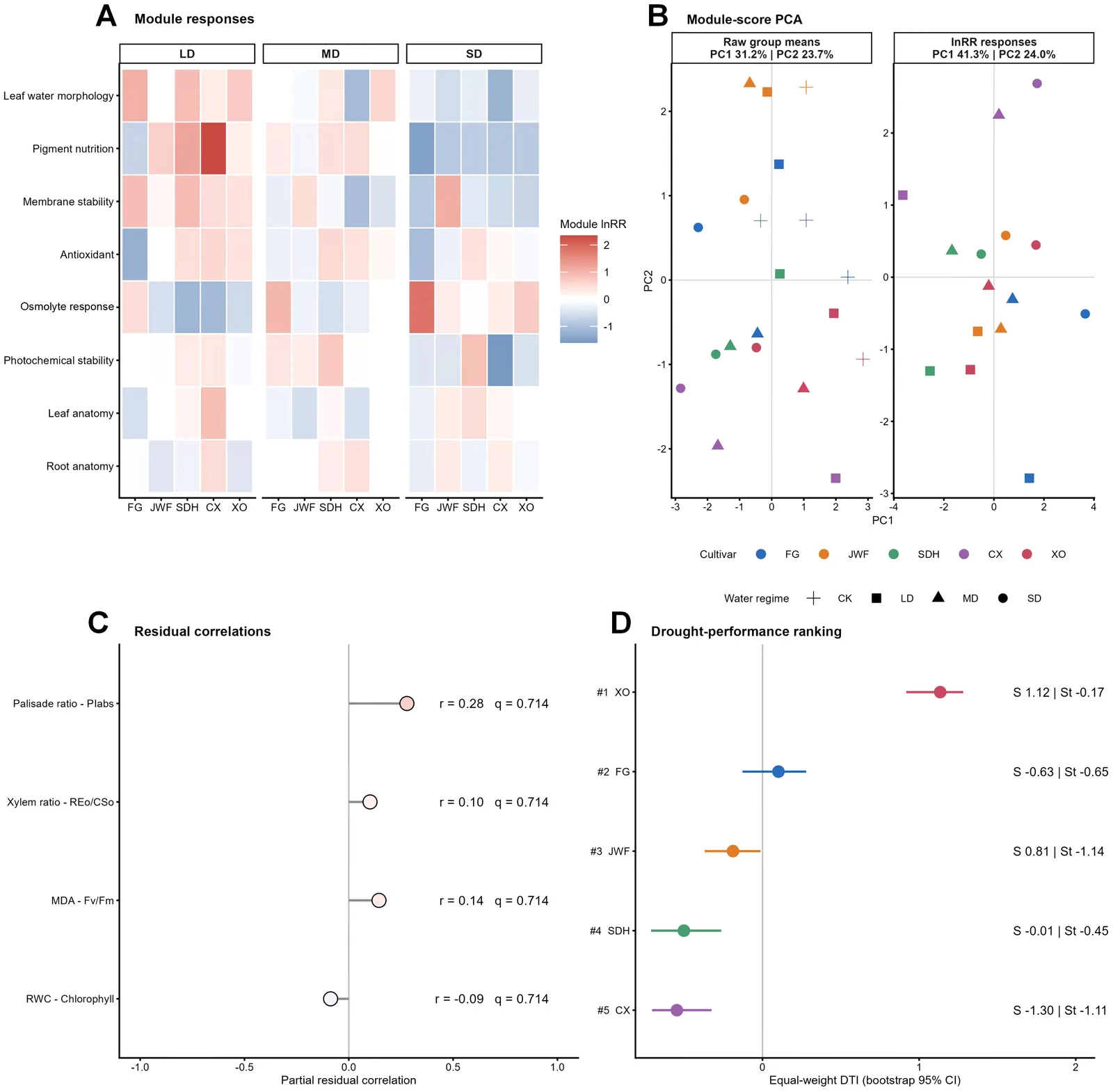
Multi-module drought responses, descriptive ordination and relative integrated performance. Cultivars are FG, Fen Guan; JWF, Jing Wang Fen; SDH, San Dian Hong; CX, Cai Xia; and XO, Xiao Ou. Treatments are CK, control (65% MWHC); LD, mild drought (45%); MD, moderate drought (25%); and SD, severe drought (5%). **(A)** Eight *a priori* nonredundant module scores for lnRR under LD, MD and SD relative to CK. Traits were standardized across 15 cultivar × drought-treatment means before equal-weight averaging. Positive membrane-stability scores denote smaller REC and MDA responses; anatomical modules retain response direction. **(B)** Descriptive principal component analysis (PCA) of eight standardized modules for 20 raw means and 15 drought lnRR means. PCA was not used for inference, causality, trait selection or DTI weighting. **(C)** Severe-drought performance (S = DTI under SD) versus cross-gradient relative-performance consistency (CRC = −SD of DTI across LD, MD and SD). Intervals are biological-replicate bootstrap 95% percentile intervals. CRC closer to zero denotes a more consistent relative position, not environment-independent stability. **(D)** Overall DTI from four equally weighted functional-maintenance modules with 95% percentile intervals and probabilities of ranks 1–5 from 2,000 plant-level bootstrap resamples. Biological n = 3 independent replicates per cultivar × treatment combination, with three plants contributing to each replicate. Plant-level measurements were averaged within each biological replicate before calculation of treatment means, module scores, and DTI values; biological replicates were the units used for bootstrap resampling. DTI represents relative performance among these five cultivars under the conditions tested and is not an absolute drought-tolerance threshold.

In the PCA based on eight modules derived from raw group means, PC1 and PC2 explained 30.6% and 23.6% of the variation, respectively (54.2% cumulatively). Pigment/NBI (loading = 0.534) and leaf water/function (loading = 0.590) had the largest absolute loadings on the two axes. In the lnRR-module PCA, PC1 and PC2 explained 36.7% and 22.8% of the variation, respectively (59.5% cumulatively); osmolyte response (loading = 0.546) and leaf water/function (loading = 0.593) had the largest absolute loadings (**Figure 8B**). Both PCAs were descriptive only.

Point estimates of overall DTI under the primary scheme were 0.346 for XO (95% CI, 0.239–0.500), 0.019 for FG (95% CI, −0.091–0.184), −0.038 for SDH (95% CI, −0.270–0.071), −0.142 for CX (95% CI, −0.316–0.070) and −0.185 for JWF (95% CI, −0.355 to −0.043) (**Figures 8C, D**). S denotes DTI under SD, and CRC denotes consistency across LD, MD and SD. XO had the highest S (0.548) and the CRC closest to zero (−0.205). XO ranked first in 99.9% of bootstrap resamples among the five cultivars under the present experimental conditions. Under the primary scheme, the probabilities of ranking fifth were 59.9% for JWF and 21.6% for CX. The 95% CI for the pairwise difference between XO and every other cultivar excluded zero, whereas intervals for FG–SDH, FG–CX, JWF–SDH, JWF–CX and SDH–CX included zero. XO ranked first by point estimate under all four sensitivity schemes. Ranks of CX and JWF varied among schemes, and correlations of schemes B, C and D with A were 0.900, 0.800 and 0.700, respectively. The corresponding bootstrap medians were 0.700, 0.800 and 0.700. Sensitivity results are presented in **Supplementary Figure 2** and **Supplementary Table 3**. An absolute second-to-fifth ranking was therefore not reported.

## 4 Discussion

### 4.1 Leaf water and functional traits, pigment retention and membrane stability underpin cultivar differences in drought tolerance

Severe drought reduced leaf relative water content (RWC) and chlorophyll index and increased relative electrolyte conductivity (REC) in all five cultivars. These endpoint responses identify leaf hydration, pigment status and membrane integrity as drought-sensitive processes. Comparable changes have been reported in herbaceous and tree peony, and natural dry-down altered SPAD and leaf traits in tree peony (Zhao et al., 2019; Li et al., 2020; Luan et al., 2023). Transcriptomic work has also identified drought-responsive genes associated with osmolyte metabolism, antioxidant defense and photosynthetic processes (Zhao et al., 2019). Because these processes are commonly measured separately, their relative contributions to cultivar performance have remained difficult to compare. The present study extends earlier observations by resolving cultivar differences within a common graded-water experiment and by integrating retained function with the uncertainty of the resulting ranking.

Cultivars differed in both proportional response and absolute status. SDH had the smallest RWC decline under SD, indicating comparatively strong water retention relative to its CK. XO showed a significant NBI decline, yet retained the highest absolute NBI under SD. These findings describe different properties: stability relative to a cultivar-specific control and the functional level reached after stress. A smaller proportional decline can coexist with a modest absolute value, whereas a high endpoint value can remain after a measurable decline from CK. The two forms of evidence should therefore not be treated as equivalent. CX combined the largest RWC decline with the greatest REC increase, identifying dehydration and membrane leakage as prominent constraints under the conditions tested.

Leaf area, SLA and LDMC did not differ from CK under SD. At the 30-d endpoint, physiological changes were therefore more evident than remodeling of leaf size or dry-matter allocation. A natural dry-down study reported smaller and thicker peony leaves (Luan et al., 2023), and the contrast may reflect drought imposition, duration, dehydration rate, leaf position or genotype. Leaves formed during sustained water limitation may acquire altered morphology through development, whereas already expanded leaves can respond first through water loss and functional decline. Because SLA and LDMC responses depend on species, plant functional type and environment (Poorter et al., 2009; Pérez-Harguindeguy et al., 2013), their stability here should not be generalized beyond this treatment period and developmental stage. The conservative Dunnett adjustment may also have limited detection of small or variable effects.

REC increased in all cultivars under SD, whereas MDA increased only in FG and decreased in JWF and CX. Membrane permeability and the measured lipid-peroxidation product were therefore not tightly coupled. MDA depends on lipid-peroxidation rate, metabolite turnover, developmental state, sampling time and analytical interference (Hodges et al., 1999; Jambunathan, 2010). The present endpoint data cannot distinguish among these explanations for lower MDA in JWF and CX. Its decline in CX did not indicate less membrane injury because REC increased most strongly in that cultivar. At this endpoint, REC provided the more consistent evidence of membrane disruption, while MDA remained a complementary oxidative-status marker rather than a stand-alone measure of cultivar injury.

### 4.2 Cultivar-specific osmotic and antioxidant responses reveal distinct compensatory patterns

Proline (Pro) and soluble sugars (SS) increased in all cultivars under SD, demonstrating a shared osmolyte-accumulation response. Peony studies have likewise linked drought with proline biosynthesis, sugar metabolism and related pathways (Zhao et al., 2019; Li et al., 2020; Luan et al., 2024). Functional analyses of P5CS and the PoWRKY69–PoVQ11 module further support roles for proline or fructose accumulation in Paeonia drought responses (Luan et al., 2024). These solutes may contribute to osmotic-potential regulation, macromolecular stabilization, carbon allocation and redox buffering (Verslues and Sharma, 2010). However, osmotic potential and quantitative solute contributions were not measured. The results therefore support osmolyte accumulation and metabolism associated with osmotic adjustment, not direct quantification of actual osmotic adjustment.

Solute accumulation did not map directly onto integrated performance. FG showed a 457.2% increase in Pro with increases in SS and soluble protein, but did not rank highest for retained pigment status, membrane stability, PSII performance, root structure or DTI. Strong accumulation may be protective through osmotic and molecular stabilization, but can also reflect a greater compensatory burden after substantial cellular stress (Verslues and Sharma, 2010). SDH retained more leaf water and PSII photochemical performance with a smaller Pro increase, whereas XO combined osmolyte accumulation with broader functional maintenance. This contrast illustrates why response amplitude and retained function were analyzed separately. FG is therefore best described as showing a strong osmolyte-response strategy rather than proven superior osmotic adjustment.

SOD, POD and CAT did not increase in concert. CAT increased only in CX, SOD only in SDH, and POD declined in FG, SDH and CX. This heterogeneity argues against a single antioxidant-enzyme configuration across Paeonia and may reflect enzyme identity, stress duration and sampling time (Zhang et al., 2019; Zhao et al., 2020, 2021). Some tree-peony studies reported broader increases in antioxidant-enzyme activity, but differences in tissue, drought progression and sampling endpoint limit direct comparison. Activity at the endpoint may also be affected by enzyme inactivation, protein synthesis and substrate availability. APX, GR, the AsA–GSH cycle and non-enzymatic antioxidants were not measured but can contribute to redox control (Foyer and Noctor, 2005; Miller et al., 2010). Increased SOD in SDH covaried with PSII maintenance, but direct ROS measurements were unavailable. CAT induction in CX co-occurred with dehydration and membrane leakage, while XO achieved the highest DTI without strong induction of these enzymes. The data therefore do not support the assumption that larger activity changes necessarily indicate more effective redox protection. Antioxidant activity was interpreted as response strategy rather than an automatically beneficial DTI component.

### 4.3 Cultivar-specific PSII photochemistry distinguishes compensatory adjustment from functional maintenance

Chlorophyll-fluorescence variables did not decline uniformly under drought. Under SD, SDH showed increases in Fv/Fm, ETo/RC and ETo/CSo; JWF showed increases in Fm, Fv and TRo/CSo; and CX showed higher Fo and TRo/CSo but lower REo/RC. PIabs in CX declined by 30.1%, although the adjusted test did not reach significance. Fluorescence responses depend on genotype, treatment duration, leaf age and protocol (Oukarroum et al., 2007; Baker, 2008; Stirbet and Govindjee, 2011; Kalaji et al., 2014). A natural dry-down study reported broader fluorescence declines in peony, indicating that drought progression and measurement timing can materially affect the profile (Luan et al., 2023). The cultivar-specific patterns here therefore describe different PSII photochemical responses rather than a uniform trajectory.

SDH combined the smallest RWC decline with the highest photosystem-stability score. Its Fv/Fm and electron-transport responses support maintenance of maximum PSII photochemical potential and electron-transport characteristics under SD. Fv/Fm represents the maximum quantum yield of dark-adapted PSII, while ETo/RC also depends on active reaction-center number and energy partitioning. The accompanying ETo/CSo response indicates that transport flux per excited leaf cross-section did not decline appreciably. These variables do not demonstrate increased net photosynthesis. Chlorophyll fluorescence cannot substitute for stomatal conductance, intercellular CO_2_ concentration or net CO_2_ assimilation, which may remain limited during drought (Lawlor and Cornic, 2002; Flexas et al., 2004; Chaves et al., 2009; Pinheiro and Chaves, 2011). The evidence therefore supports PSII photochemical maintenance in SDH within the measured endpoint.

In CX, higher Fo and TRo/CSo combined with lower REo/RC, consistent with a mismatch between excitation-energy trapping and downstream electron transport. This pattern covaried with lower RWC, higher REC and a low photosystem-stability score, but it does not establish causality. JWF responses were concentrated in trapping and primary electron transport without a comparable downstream advantage, whereas most fluorescence variables changed little in XO, consistent with endpoint photochemical stability. Differences among cultivars may also depend on drought imposition, dark-adaptation duration and normalization per reaction center or leaf cross-section (Oukarroum et al., 2007; Živčák et al., 2008; Stirbet and Govindjee, 2011; Kalaji et al., 2014; Osório et al., 2014).

### 4.4 Organ- and cultivar-specific leaf-root anatomical plasticity

Under SD, leaf thickness decreased in FG, JWF and SDH but did not change significantly in CX or XO. This cultivar specificity is consistent with the context dependence of leaf structural responses and their association with tissue density and resource investment (Poorter et al., 2009; Pérez-Harguindeguy et al., 2013; Scoffoni and Sack, 2017). Huang et al. reported thicker leaves in three herbaceous peony cultivars after natural dry-down (Huang et al., 2026). Leaves developing during water limitation may become thicker through developmental acclimation, whereas expanded leaves can become thinner through tissue contraction. Differences in drought progression, leaf age, sampling position and fixation may therefore contribute to the contrast. The present measurements describe endpoint structure and cannot resolve developmental acclimation from tissue contraction.

Proportional tissue traits require interpretation alongside total leaf thickness. In SDH, palisade proportion increased while total thickness declined, which need not represent an absolute increase in palisade tissue and may reflect unequal contraction among tissues. Higher epidermal proportions in FG and JWF may likewise include denominator effects from thinning. No cultivar showed a significant SD response in spongy proportion or the palisade-to-spongy ratio, providing no evidence for a shared reorganization of mesophyll proportions. The smaller structural changes in CX and XO indicate retention relative to CK, but do not establish whether retention resulted from favorable baseline anatomy or limited plasticity.

Root anatomical responses were strongly cultivar dependent. FG showed reductions in root diameter, periderm and phloem thickness, consistent with contraction across several measured regions. JWF and XO showed increases in root diameter, phloem or xylem-region dimensions, while xylem proportion increased in JWF. CX responded mainly through cambium thickness and SDH changed little overall. Root drought responses can involve trade-offs among soil exploration, carbon investment and conductive-tissue allocation (Comas et al., 2013; Lynch, 2013, 2018; Strock and Lynch, 2020; Sun et al., 2022). Greater vascular-region allocation could be relevant to transport capacity, while greater peripheral investment could affect protection and construction cost. However, the functional consequence depends on vessel architecture and water availability along the root system, neither of which was measured. The present measurements indicate structural or vascular allocation responses, but they do not measure vessel lumen diameter, density, wall thickness, embolism vulnerability or hydraulic conductance. Increased xylem-region width in JWF and XO therefore cannot be equated with improved hydraulic efficiency or safety. Studies linking lignification and cell-wall reinforcement to Paeonia drought responses provide useful hypotheses for future direct tests, but do not convert the anatomical dimensions measured here into hydraulic evidence. Across organs, XO showed broad structural retention, SDH emphasized leaf-water and PSII maintenance, CX showed a localized cambial response, and FG combined structural contraction with strong osmolyte accumulation.

### 4.5 Multi-module integration and DTI separate response magnitude from integrated drought performance

The raw-module PCA described functional states reached under treatment, whereas the lnRR-module PCA described change relative to each cultivar’s CK. Pigment/NBI and leaf water/function had the largest loadings in the raw ordination, while osmolyte response and leaf water/function had the largest loadings in the lnRR ordination. The first two axes explained 54.2% and 59.5% of variation, respectively. FG illustrates the distinction because its large osmolyte response was not accompanied by the highest integrated maintenance score. These ordinations summarize different views of the same treatment matrix, and a large response is not equivalent to a favorable endpoint. The descriptive associations distinguish absolute state from response magnitude but neither establish causal links nor determine DTI weights.

Within this trait framework, XO combined pigment-NBI, water, PSII and root-structural maintenance; SDH emphasized leaf-water and PSII maintenance; and FG showed strong osmolyte accumulation. Localized CAT and anatomical responses in CX did not coincide with comparable water or membrane maintenance, whereas JWF emphasized root structural adjustment without a consistent whole-plant advantage. These profiles help separate localized compensation from coordinated maintenance across modules. However, the labels are working summaries of the measured response patterns, not fixed or mutually exclusive physiological types. Their expression may change with environment, developmental stage, substrate or treatment duration.

The experiment-specific DTI provided the strongest relative support for XO. Its point estimate was 0.346, its 95% CI excluded zero, and it ranked first under all four sensitivity schemes. XO ranked first in 99.9% of bootstrap resamples among the five cultivars under the present experimental conditions. The bootstrap value quantifies within-experiment ranking uncertainty, not the probability of universal drought tolerance. Pairwise intervals for XO versus each other cultivar excluded zero, whereas most intervals among FG, SDH, CX and JWF included zero. The ranks of CX and JWF also changed across sensitivity schemes. By combining *a priori* functional-maintenance modules with confidence intervals, rank probabilities and sensitivity analyses, the framework avoids treating a point ranking as error free and exposes dependence on module choice. This uncertainty-aware integration, rather than any individual physiological or anatomical assay, is the principal methodological advance of the study. Equal weighting remains an explicit assumption, but the sensitivity schemes show which conclusions persisted when module composition changed. For the intermediate cultivars, differences in response strategy are therefore more defensible than a fixed second-to-fifth ordering.

### 4.6 Implications, limitations and priorities for validation

The results identify XO as the leading candidate for subsequent drought-tolerance validation and potential evaluation as a breeding parent. SDH and FG provide contrasting material for investigating leaf-water and PSII maintenance versus strong osmolyte responses, while CX and JWF illustrate localized compensation and discordant leaf-root performance. These contrasts may be useful for hypothesis-driven validation, but they do not establish breeding value. This interpretation is limited to a single 30-d greenhouse pot experiment, one substrate, one developmental stage and predefined nominal MWHC targets. No independent continuous substrate-water trajectory was analyzed, and field performance may differ with climate, soil distribution, drought timing and perennial carry-over effects. The DTI is therefore a comparative index within this experiment, not an externally validated threshold.

The study compared endpoint responses and did not resolve drought onset, sustained-treatment dynamics or recovery after rewatering. Leaf water potential, gas exchange, ABA, reactive oxygen species and direct hydraulic traits were not measured. Consequently, stomatal and non-stomatal limitations cannot be separated, antioxidant activity cannot be linked causally to injury, and anatomical dimensions cannot establish hydraulic function. SOD, POD and CAT also represent only part of the antioxidant network, while xylem-region width does not describe vessel architecture or embolism risk. The absence of rewatering data also prevents assessment of whether apparent maintenance during stress translated into faster or more complete recovery. Bootstrap resampling captures uncertainty among the biological replicates studied here, but not variation among years, sites, substrates or production systems. Independent validation should therefore prioritize contrasting environments, drought-rewatering designs and direct functional measurements relevant to the proposed mechanisms. For a perennial ornamental, longer-term evaluations should also consider recovery, flower quality and renewal growth.

## 5 Conclusions

Graded drought altered leaf hydration, pigment status, membrane permeability and osmolyte accumulation in all five cultivars, whereas antioxidant, PSII photochemical and leaf-root structural responses were cultivar specific. FG showed strong osmolyte accumulation, SDH maintained leaf water status and PSII electron transport, CX showed pronounced dehydration and membrane leakage despite localized responses, and JWF emphasized root structural adjustment. XO showed the most coordinated maintenance of pigment-NBI status, photosystem performance and root vascular structure.

Within this 30-d greenhouse pot experiment, the DTI point estimate, confidence interval, rank probability and sensitivity analyses identified XO as the leading candidate for subsequent drought-tolerance validation. XO ranked first in 99.9% of bootstrap resamples among the five cultivars under the present experimental conditions, but this does not establish universal or field-level superiority. The conclusion is bounded by the single environment, fixed substrate, endpoint design and absence of gas exchange, water potential, direct hydraulic and rewatering measurements.

## Data Availability

The datasets generated for this study are available from the corresponding author upon reasonable request. Requests to access these datasets should be directed to Baosheng Shi, baoshengshi@163.com and Yanhua Pan, panyh2006@163.com.
