# Supplementary material for: Integrated physiological, photochemical and anatomical responses reveal divergent drought-response strategies among herbaceous peony cultivars
Source: Front Plant Sci. 2026 Sep 14;17:1958940. doi: 10.3389/fpls.2026.1958940 (PMC13616670; doi:10.3389/fpls.2026.1958940)
Supplement: Supplementary file 1 [file SupplementaryFile1.zip › Supplementary Material/Table 1.DOCX]

**Supplementary information**

S1. Experimental design and overall factorial effects

The experiment included five peony varieties—FG, JWF, SDH, CX, and XO—as well as four moisture treatments: CK (65%), LD (45%), MD (25%), and SD (5%). Biological replicates and technical measurements were analyzed separately: for microstructure, 3 biological replicates × 5 fields of view per plant; the actual n values for the remaining modules are shown in Figure S1 and the statistical workbook. This section is provided as supplementary information.

After fitting cultivar × water models to each of the 58 traits dynamically identified by trait_inventory, 51 traits showed a main effect of cultivar, 35 showed a main effect of water content, and 29 showed a cultivar × water interaction, all reaching a BH-FDR q < 0.05. Only the total numbers are reported here; no explanation is provided for the physiological mechanisms underlying the interactions. F, P, q values, transformed scales, and diagnostics for each trait are provided in the Supplementary Statistical Tables.

Figure S1 | Experimental design and analytical coverage. Drought treatments were CK = 65%, LD = 45%, MD = 25% and SD = 5%. Biological replicate counts are shown by measurement module. Microscopy quantification used three independent plants per group and five technical fields per plant; technical fields were averaged before inference.

## Table S1. Omnibus factorial effects after BH-FDR

| **Effect** | **Traits tested** | **q < 0.05** | **Location of full results** |
| --- | --- | --- | --- |
| Cultivar | 58 | 51 | 09_Supplementary_Statistical_Tables.xlsx |
| Water | 58 | 35 | 09_Supplementary_Statistical_Tables.xlsx |
| Cultivar × water | 58 | 29 | 09_Supplementary_Statistical_Tables.xlsx |

Supplementary Figure S2 | Sensitivity of the drought tolerance index to module definitions. a, Overall DTI and biological-replicate bootstrap 95% percentile intervals for the primary four-module scheme (A), deletion of the structure module (B), REC-only membrane stability (C), and the alternative absolute-anatomy indicator set (D). b, Spearman correlations among point-estimate cultivar ranks. Complete bootstrap rank correlations, pairwise cultivar differences and rank probabilities are provided in Supplementary Table S3.
